# Teaching clinical hematology and leukocyte differentiation in veterinary medicine using virtual patients

**DOI:** 10.3389/fvets.2023.1163927

**Published:** 2023-09-19

**Authors:** Hannah Marahrens, Matthias Gerhard Wagener, Elisabeth Schaper, Jana Zintl, Frederik Kiene, Martin Ganter

**Affiliations:** ^1^Clinic for Swine and Small Ruminants, Forensic Medicine and Ambulatory Service, University of Veterinary Medicine Hannover, Foundation, Hannover, Germany; ^2^Center for E-Learning, Didactics and Educational Research, University of Veterinary Medicine Hannover, Foundation, Hannover, Germany

**Keywords:** virtual patients, digital teaching, hematology, veterinary medicine, blood cell count, leukocyte differentiation

## Abstract

Due to contact restrictions imposed because of the COVID-19 pandemic, we created a novel digital course on the Moodle learning platform for winter term in 2020. In the clinical pathology course (CPC) with hematological content, third-year students were able to work independently on 10 extra digital cases of internal medicine involving eight different animal species as a compensation for the reduction in traditional microscopy exercises. Each case presented was initiated using an anamnesis, also the participants to generate a differential blood count based on digitized leukocytes, previously been photographed using a microscope camera. The cases were successive and increased in complexity, for example through the increase in the number of different cell types to be differentiated. The participants had the opportunity to evaluate the course through a final module to rate user-friendliness and acceptance. The total results of the participants in 2021 were analyzed descriptively, focusing on success rates, time spent on the tasks, and number of attempts. A total of 237 (= 96%) of 247 students completed all cases, each assessing 1033 photographed blood cells in sum. The mean processing time was 22.48 min for a differentiation and the students spent an average of 1.48 attempts on it. A voluntary feedback form was completed by 192 (= 78%) students, with more than 95% rating the course positively in 12 evaluation questions, and 29 of 33 comments (= 87.88%) providing positive statements in a comment box. Suggestions for improvement primarily included more explanations on erythrocyte morphologies, followed by adjusting the difficulty level and improving the presentational set-up. Slight improvements in results, time spent on processing the tasks, and the number of attempts indicated an achievement of routine and confidence during the course and were associated with an increase of competency. The positive feedback showed a high acceptance of the digital format and students evaluated the course as improving the quality of teaching when combined with practical exercises.

## 1. Introduction

In addition to the clinical examination, the compilation and interpretation of diagnostic laboratory findings is essential for achieving a diagnosis and deciding on appropriate treatments. In daily veterinary practice, blood tests are routinely performed with the assistance of automated systems. Due to the advances of the last decades, new and refined systems provide much more precise results and increased specificity, especially in the field of small animal medicine (1–4). Despite this, species-specific differences in blood composition are still sources of inaccuracy and can lead to discrepancies in laboratory results. This is particularly evident in the field of hematological diagnostics, concerning different cell morphologies of leukocytes and erythrocytes depending on the species, as well as in uncertain findings on platelet fractions, which vary greatly in their reliability (2, 4–6). Pathological changes in the blood cell count composition also continue to pose a challenge for automated systems. In addition, automated systems have failed so far to reliably detect certain pathological findings such as hemoparasites, bacteria or morphological changes in blood cells due to genetic defects, mineral-/vitamin-deficiencies, or other causes. Skills in traditional manual laboratory techniques enable examiners to prepare blood cell counts by hand as a control when required, including situations where analytical equipment is not available. Manual procedures are also commonly used as a gold standard for quality controls (QC) (7). Therefore, the competence to have a critical view on automated laboratory findings and the ability to check results manually are defined as learning objectives in teaching basic hematology at veterinary schools in Germany. To implement this principle, third-year students at the University of Veterinary Medicine Hannover, Foundation (= TiHo), practice basic skills in a weekly course run by the Clinic for Swine and Small Ruminants as a part of their training in clinical pathology (clinical pathology course = CPC). Learning manual techniques such as simple microscope handling and routine hematological procedures including packed cell volume (PCV) and photometric hemoglobin determination and preparation of blood smears and manual leukocyte counts (WBC = white blood count) formed the first part of the CPC. It also included repeated training in the manual differentiation of leukocytes (= dWBC) by microscopy, where the students were supported by the lecturers and laboratory personnel. Due to the onset of the COVID-19-pandemic, the institution had digitized parts of its teaching. To compensate for the omission of in-person hematological microscopy exercises, the institution developed a module on the learning management system Moodle (TiHoMoodle). The face-to-face classroom sessions of the course in compliance with the prescribed hygiene measures had to be reduced and accompanying lectures were given online. Seven training sessions (14 h) addressing clinical hematology were reduced to one session. Since recognizing blood cell morphologies requires routine, the idea was to teach leukocyte differentiation in the form of clinical cases, as the teaching format using virtual patients has generally found a high level of acceptance among students in human medical education (8) and in veterinary education (9–13). Moodle had already been used in digital medical education in different institutions before COVID-19 due to technological advances and the increasing importance attached to digital teaching in addition to in-person lectures and exercises (14–19). The software provides a suitable platform, as the content can be integrated in a combination of different question types such as text questions, single-choice or multiple-choice tasks, assignment questions, and picture questions as well as free-text task forms (20). At various points, the lecturers can provide information in the form of scripts or slides, and helpful hints can be integrated at certain key points. In addition, the possibility of direct communication between students and teachers exists through the optional establishment of a forum or chat. Finally, the participants have the opportunity to provide feedback in an optional pre-configured final module in the form of rating scales or free-text answers. Those modules can still be manually adjusted by the lecturers according to their own interests (21). The Moodle course on hematology (= MC) was intended to provide an interactive opportunity to apply and practice the previously learned hematological content, to increase flexibility of the participants' schedules, and to encourage independent working. The performances are standardized as all students interacted with the same blood cells in the identical clinical cases and thus are comparable as the students' results could be exported and statistically evaluated. Our study objectives were the investigations of the changes in test results, the amount of time spent on the tasks, and the number of attempts each student made during the course. In addition to presenting the results, this paper illustrates the student's perception and the usage of the created MC next to reflecting on the observed advantages and disadvantages of the course as a possible solution for digital teaching in hematology.

## 2. Materials and methods

### 2.1. Concept

Attendance at the CPC was compulsory for third-year students in the winter term. Participation in the MC was voluntary if all practice sessions had been attended in full. One date of absence of attendance hours was replaced by work on five cases on the Moodle platform. A total of two dates of absence were approved for credit. After the students had been introduced to the concept and had practiced the basics of hematological procedures in the first attendance hour of the CPC, they were able to start the MC “Exercises on hematology as a supplement to the course in clinical pathology”. The course included 10 cases of different species based on pre-existing hematological data of clinic's patients and the corresponding archived blood smears. The selected species were based on animal species that are common in veterinary practice in Germany, such as cats, dogs, cattle, goats, horses, pigs, or sheep. Thematic contents dealt exemplarily with common or practice-relevant hematological cases. The basis of all cases was the differentiation of 100 leukocytes previously photographed in oil immersion with a microscope camera using 1000-fold magnification (microscopy technique: Axiolab 5 light microscope with Axiocam 208 color camera; Carl Zeiss Meditec AG, Jena, Germany). Settings of picture quality were controlled with the assistance of the software program ZEN 3.0 blue edition.Ink (Carl Zeiss Meditec AG) and the captured images were subsequently cropped using Microsoft^®^ Paint 3D program (Microsoft Corporation, CA, USA), so that the images had a uniform size of 1500 × 1500 pixels and the cells were located in the center of the image. The blood smears were stained using standard hematological staining in accordance with Pappenheim (22), consisting of May-Grünwald and Giemsa staining solutions (manufactured by Merck KGaA, Darmstadt, Germany). To encourage the students to make internal medical interrelationships and to avoid monotonous working on blood cells, the preparation of the dWBC was supplemented by further tasks to complete a clinical case with a hematological focus. Therefore, the results of the blood tests should provide indications for further diagnostics. The main question types were single-choice questions to recognize cells, multiple-choice questions to summarize areas, and free-text entries to indicate calculation results. Due to default settings set by us, the students had an unlimited number of attempts per task, with the best attempt being scored. The learning success and the increase in self-confidence were to be examined by comparing the results of the individual cases based on the success rates, the differentiation time, and the number of attempts per case. To obtain the results of the success rates in differentiation, the number of correctly recognized cells was divided by the total number of cells presented per student, with 1.00 being the maximum achievable result. A differentiation task was passed if 80% of the cells were correctly identified. For didactic reasons, there was the possibility to directly check the correctness of the answer, without the ability of a subsequent improvement in the context of a started attempt at the respective task. There was no time limit for completion of any of the tasks and the students could complete the cases at any time in the course of the lecture period of the winter term from the second week onwards (lecture term: October 11, 2021—January 28, 2022) and also one week thereafter. Due to the thematic dependencies, the order of the cases and the tasks they contained were predetermined. A task could only be started when the previous one had been completed. The students had the opportunity of giving feedback in a final module and were always able to contact the lecturers via text messages.

### 2.2. Course structure

At the beginning of the MC, introductory slides with instructions explained the principles and structure of the course, whereby it was also recommended to work on the individual cases after completing the corresponding lesson in the CPC. Learning objectives were defined and references to further literature were provided. This was followed by information on data protection, with consent being obtained from all participants by means of a single-choice question. In order to repeat hematological contents from the lecture and from the attendance hours of the laboratory course, the next section included slides with information, reference values, and illustrations of the different blood cell morphologies. This also contained an upload with a scheme used for morphological differentiation of leukocytes in tabular form, which the students had to fill out manually for each differentiation task in order to calculate leukocyte percentages later and to be able to create their dWBC.

**Figure 1:**
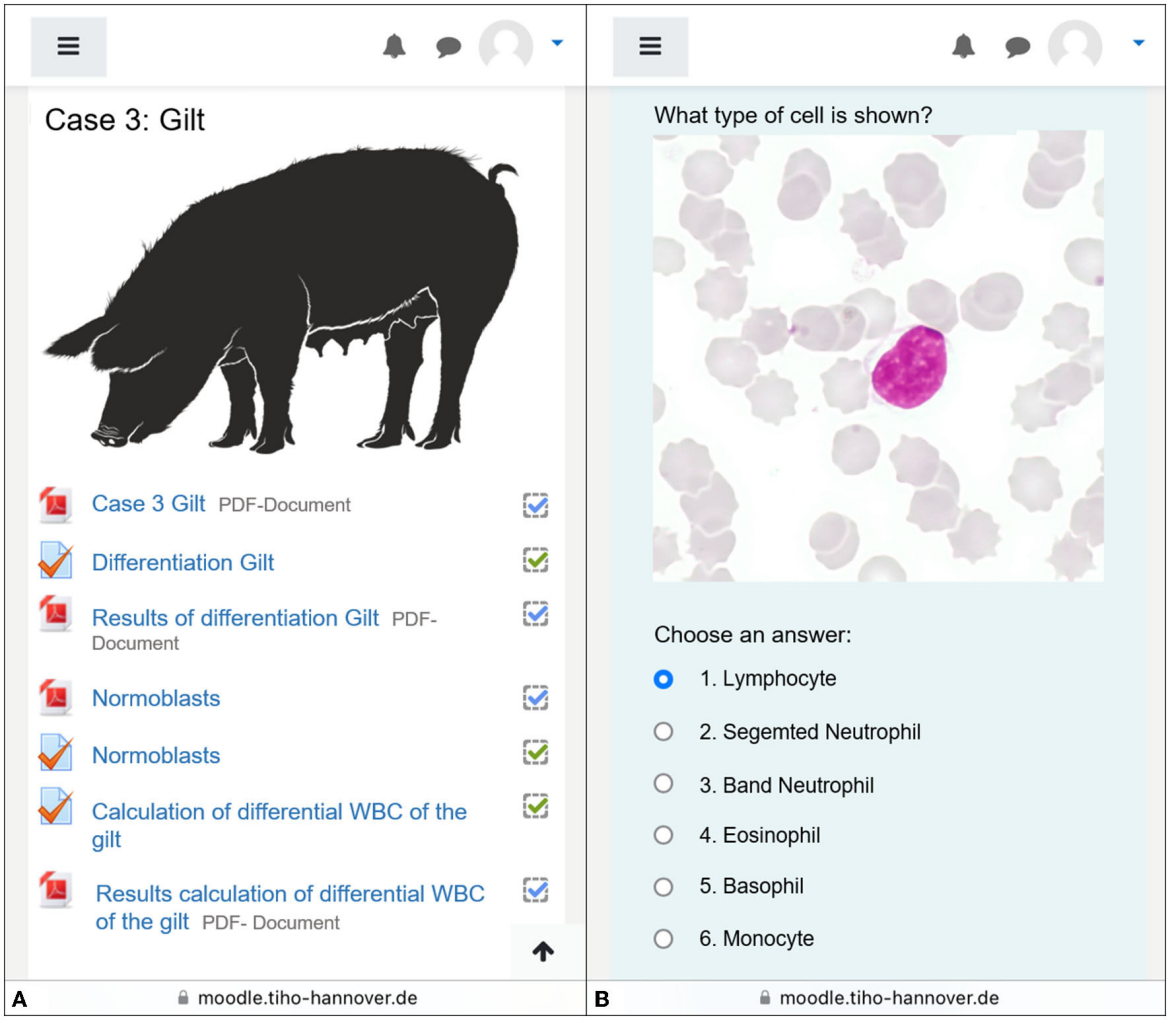
Screenshot taken by smartphone working on case 3. **(A)** The exemplary overview of some segments of the case. Information given via PDF-documents, followed by tasks. Each section is individually clicked during processing. The processing status can be seen on the right-hand side by the ticks. **(B)** A lymphocyte in the differentiation task. Students have determined all the leukocytes shown individually. Illustration of animal species found on Pixabay, open source. Source for the picture of the gilt: https://pixabay.com/de/service/terms/.

The subsequent cases were structured linearly (Figure 1A) into three parts according to the same principle:

1. Introduction
2. White blood count
3. Red blood count.

**Figure 2:**
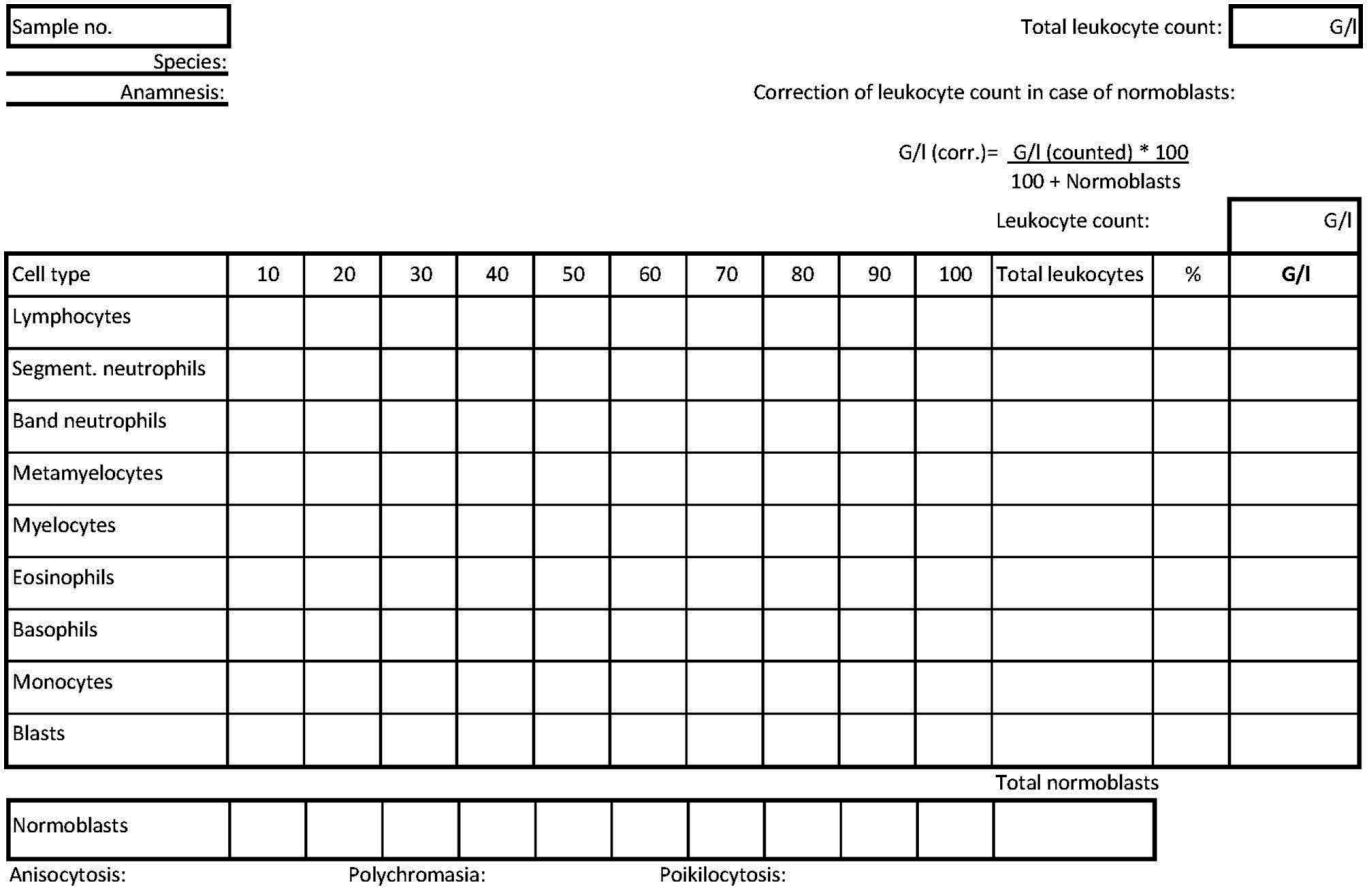
Scheme manually filled out during differentiation tasks. Each column contains the count of 10 leukocytes. At the end, the percentages and thus the dWBC can be determined. All possible leukocyte forms are listed. The counting of possible normoblasts is in a separate line. The formula for correcting the total leukocyte count using the normoblast count is given in the upper right corner based on the total leukocyte count. In addition, the findings of the erythrocyte forms are entered. There are fields for notes on the preliminary report.

The introduction was a preliminary report including anamnesis and initial findings of clinical investigations or first clinical pathological findings. The second part of each case started with the differentiation of at least 100 images of cells including 100 leukocytes using the test option in Moodle. Therefore, one single-choice task was created for each cell, in which the photographed cell had to be assigned by the students to a specific cell type (Figure 1B). One point was awarded for each correct answer, i.e., for each correct cell allocation. The cells were randomly presented to each student, while the answer choices remained the same within a case, including all cell types that could occur in the case itself. During the processing, the students manually filled out the tally chart in the differentiation scheme provided at the beginning (Figure 2). With further cases, leukocytes were expanded to include other cell types occurring in the blood smears to enable the students to distinguish normoblasts as immature, nucleated erythrocytes from leukocytes, and to recognize platelet aggregates as frequent reasons for inaccuracies in automated counters (Table 1). Based on the differentiation scheme completed in parallel to the differentiation, the subsequent task required the calculation of the absolute leukocyte numbers (G/l) and thus the results of the dWBC in the form of eight free input fields. This calculation task was kept consistent from case to case by also asking for cells that might not have been present in the respective smear and therefore had to be answered with 0.0. Since an increased total leukocyte count caused by normoblasts in error needs to be corrected (23), the information on whether normoblasts occurred within the differentiation or not was also requested by a simple Yes/No—single choice. At the end of the WBC part, a summary of the diagnoses of the previous leukocyte findings was asked for by multiple choice. A subsequent slide summarized these findings in more detail and contained initial explanations before moving on to the third part dealing with red blood count.

**Table 1:** Number of cell types occurring in the blood smears sorted by their frequency in the Moodle course in total and in individual cases.

**Cases**	**Total number**	**1**	**2**	**3**	**4**	**5**	**6**	**7**	**8**	**9**	**10**
Number of cells	1033	100	100	102	100	101	122	100	101	105	102
Segmented neutrophils	460	X	X	X	X	X	X	X	X	X	X
Lymphocytes	301	X	X	X	X	X	X	X	X	X	X
Band Neutrophils	93		X	X		X	X			X	X
Eosinophils	55	X	X	X	X		X	X	X	X	X
Monocytes	36	X		X	X	X	X	X	X	X	X
Metamyelocytes	35			X		X					
Normoblasts	26			X		X	X			X	
Basophils	11				X			X	X		
Myelocytes	8			X		X					
Thrombocyte aggregates	8						X		X	X	X

In order to assess the morphology of the erythrocytes, 10 different photographic image fields of monolayer areas of the respective blood smears, where red blood cells appeared isolated and with central paleness, were first uploaded as collective slides. In three consecutive single-choice tasks, the students assessed the conspicuousness of the erythrocyte morphologies on anisocytosis, polychromasia, and poikilocytosis with answer options “none”, “+”, “++”, “+++”, and “++++”. Based on given red blood count results, the erythrocyte indices (MCV, MCH, and MCHC) then had to be calculated, again using free input fields. Data for erythrocyte count, hemoglobin, and PCV had to be taken from the medical history at the beginning of each case for this purpose. A subsequent single-choice question related to diagnosed anemia, asking whether it was a regenerative or non-regenerative form. The section on the red blood count ended with a multiple-choice question on the character of the anemia or other findings from the red blood count. At the end of each case, a case-closing summary was provided with possible interpretations of the findings obtained from the blood count in relation to the preliminary report and to the clinical symptoms reported. An overview of thematic characteristics of the cases and their individual features is given in Table 2.

**Table 2:** List of all created cases and species of the TiHoMoodle course with their thematic content and the corresponding results of blood counts.

**Case, species**	**Thematic content**	**Blood count results**	**Peculiarities**	**Diseases addressed**
1. Donkey	Donkey with reduced feed intake, subfebrile body temperature, and intermittent nasal discharge.	Lymphocytosis.	No further tasks to facilitate the introduction of the case principle.	Chronic infectious disease of the respiratory tract.
2. Sheep	Lamb with previously reported coccidiosis, still suckling, with increased body temperature and low weight gain. Only moderate improvement after treatment.	Leukocytosis, neutrophilia. Non-regenerative, normochromic, normocytic anemia.	Hypersegmented neutrophils (more than 5 segments) = nuclear right shift (24).	Chronic purulent inflammation.
3. Swine	Grit from a farm with increased abortion and premature birth rates as well as puerperal disorders. Piglet losses have increased. The sow with purulent discharge is restless; breast complexes are clinically hardened and sensitive to pain.	Leukocytosis, lymphocytopenia, neutrophilia. Regenerative, hypochromic, normocytic anemia.	Band neutrophils, metamyelocytes, myelocytes, transitional forms, = nuclear left shift (24, 25). Normoblasts.	MMA (metritis, mastitis, agalactia). Iron deficiency, anemia. Birth-related anemia.
4. Goat	Dyspnoeic goat from a small hobby farm that began to waste away in connection with a respiratory disease.	Leukocytosis, lymphocytosis, neutrophilia, basophilia. Regenerative, normochromic, microcytic anemia.	Nuclear right shift. Small size of erythrocytes shape deviation of most erythrocytes Increased MCHC. Decreased MCV	Basophilia in connection with allergic or parasitic reactions. Spindle (like Sickle) cell anemia in goats (supposed mutation in the ß-chains of hemoglobin).
5. Alpaca	Lean female alpaca with depressed abdomen and thin, grayish feces.	Neutropenia. No deviation in red blood count.	Physiological oval shape of erythrocytes of camelids. Finely granulated neutrophils, metamyelocytes, myelocytes, transitional forms = nuclear left shift. Normoblasts.	Acute enteritis. Neoplastic changes.
6. Swine	Weaner pig from a farm with MMA problems, hemolytic anemia in the breeding sows, and enterotoxemia in weaners.	No deviation in the leukocytes from reference values. Regenerative, hypochromic, microcytic anemia.	Changes in erythrocytes, Howell-Jolly-bodies, Pitted erythrocytes. Bacterial Infections of the erythrocytes. Basophilia, normoblasts, aggregation of PLTs.	Iron deficiency anemia. Bleeding anemia. *Mycoplasma suis* infection.
7. Sheep	A 2-year-old female sheep from a flock of 200 ewes, which was conspicuous with lassitude, bottle jaw, and icteric mucous membranes.	Leukocytosis, neutrophilia, eosinophilia. Regenerative, normochromic, normocytic anemia.	Eosinophilia.	Parasitic infection of grazing animals, such as hemonchosis or fasciolosis.
8. Reindeer	A reindeer with fever (40.8°C), bilateral increased breath sounds, and pale conjunctival membranes.	Neutropenia. Regenerative, normochromic, normocytic anemia.	Inclusion bodies (morulae) in granulocytes. Howell-Jolly-bodies. Azurgranulation in lymphocytes.	*Anaplasma phagocytophilum* infection.
9. Dog	Apathetic female Dachshund with increased water intake and low-grade vaginal purulent discharge. Four weeks ago, she was in heat.	Leukocytosis, neutrophilia, monocytosis. Non-regenerative, normochromic, normocytic anemia	Nuclear left shift.	Monocytosis in dogs Pyometra
10. Cat	Outdoor cat, conspicuous by emaciation.	Leukocytosis, eosinophilia, monocytosis.	Howell-Jolly-bodies in cats. Morphologies of erythrocytes and thrombocytes in comparison to dogs.	Eosinophilia in cats. Gastrointestinal helminths in cats.

The “Peculiarities” column lists special characteristics of each case. “Diseases addressed” includes all diseases that were mentioned as parts of the diagnosis or differential diagnosis.

The course completion consisted of final slides and the subsequent feedback, which could be filled out voluntarily by the participants.

### 2.3. Feedback module

The final feedback module consisted of 14 tasks, where the participants initially made 12 ratings in compact form. The first three questions dealt with the personal experience and assessments of the individual students, including fulfillment of one's own expectations (content shown in Table 4). The subsequent questions targeted the interaction between lecturer and students by asking about the ease of communication, the definition of learning objectives, and the amount of learning material provided. The last two rating questions then focused on the technical features of the course. The students could choose between the answers: 1. strongly agree, 2. tend to agree, 3. tend to disagree, and 4. strongly disagree. Subsequently, two free-text fields were used to ask for suggestions for improvement and further comments.

### 2.4. Evaluation and statistical analysis

**Figure 3:**
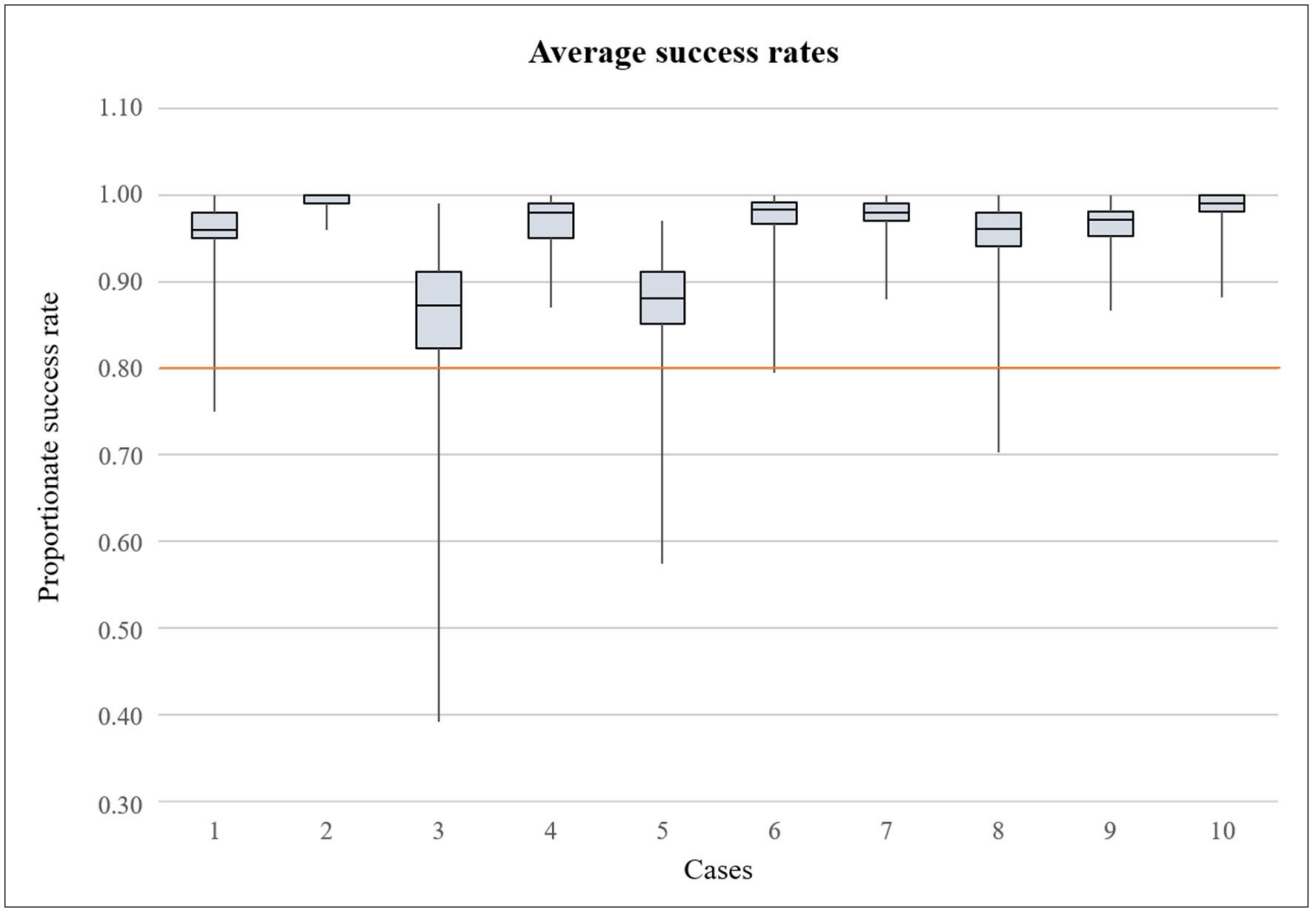
Boxplot illustrating the average success rate of leukocyte differentiation by the participants in each case. Maximum achievable score was 1.00. The orange line indicates the pass mark of 80% (0.80).

The descriptive statistics focused on leukocyte differentiation tasks, calculation of the dWBC, and the erythrocyte indices (= EI). The learning success and the change in self-confidence were to be examined by comparing the results of the individual cases based on the success rates, the differentiation time, and the number of attempts per case. To obtain the results of the success rates in differentiation, the number of correctly recognized cells was divided by the total number of cells presented per student (Figure 3). However, students could report attempts as completed even though in some cases not all cells were allocated. In addition, the second or third differentiation attempt was much better due to the assessment of the same cell images. Since the editing time was also unlimited, only data meeting the following criteria were included in the analysis in order to create more comparability:

1. Each student's first completed attempt per case to ensure improvement may be related to the practice of morphologies and not to the repeated processing of individual photographs.
2. Attempts, which were shown as “completed” in the processing status.
3. Attempts, in which at least 50% of cells were viewed and assessed.
4. Attempts in which the completion time did not exceed 60 min in differentiation or 20 min in the calculation tasks.

If first started attempts for a differentiation run were prematurely aborted (see point 2: status “in progress”), they were replaced by the second completed attempt, as this was rarely the case and the results would otherwise be statistically distorted. If the differentiation was processed in fewer than 50% of the cells but was still handed in (i.e., indicated a processing status “completed”, see point 3), we assumed that the task had been abandoned prematurely, which could also distort the results. If more than 50% of the cells had already been processed and the attempt had been submitted incomplete, we found it was an active decision to hand in the attempt. The time limits of 60 or 20 min were set according to an average consideration, as the program was sometimes not completed after task editing and the processing time was thus incorrectly extended. Results of the study objectives that did not fulfill the conditional criteria described above were sorted by coding and with the assistance of reporting filters of the Excel Pivot Table tools. In the tasks of the study objectives, the number of subtasks differed in some cases. The total number of cell-images varied from case to case, and in dWBC, six single calculations were required in case 2 and eight single calculations were required in the other cases. Therefore, the processing time was initially related to a single task in order to create comparability.

While the leukocyte fractions of the dWBC had to be calculated in all cases except case 1, the calculation of the erythrocyte indices was required in eight cases (cases 2, 3, 4, 6, 7, 8, 9, and 10). The statistical procedures were the same as those used to evaluate the differentiation results, whereby the time criterion for inclusion in assessment was 20 min in both tasks. The tasks remained the same, as these tasks were simple calculations where only the values changed.

Descriptive statistical analysis with calculation of mean values (= MV) and standard deviations (= SD), charts, and graphs were created using Microsoft^®^ Office Excel 2016 (Microsoft Corporation).

The results of the feedback module for the evaluation of the course by the participants were also processed using Microsoft^®^ Excel. The ratings were analyzed descriptively and presented quantitatively. Content of the free-text responses were evaluated with the assistance of co-workers from the Center for E-Learning, Didactics, and Educational Research (ZELDA) of the University of Veterinary Medicine Hannover Foundation for analyzing the thematic content qualitatively and quantitatively. Therefore, sub-categories (“codes”) were formed by reading the responses together, which were further combined to “themes” depending on the topic as an existing valid approach for evaluating surveys in accordance with Brown and Clarke (26). The creation of categories based on thematic content of the participants' answers only was related to the inductive categorization in accordance with Mayring (27). The procedure had already been successfully used at the TiHo (12).

### 2.5. Ethical statement

This study was conducted in accordance with the ethical standards of the University of Veterinary Medicine Hannover, Foundation. The project was reviewed and approved by the Data Protection Officer of the university. The participants consented to the processing of their data at the beginning of the MC with access to the data protection notice containing the EU General Data Protection Regulation of 2018. All results were anonymized and processed in accordance with the data protection regulation of the university.

## 3. Results

### 3.1. Casework results

In total, 247 students dealing with hematological diagnostics for the first time as part of their studies participated in the MC enrolled in the third year CPC. The course was completed by a total of 237 (= 96%) students (Table 3). Within the possible processing time, the MC was processed in a minimum of 0.30 and a maximum of 100.02 days. The mean value (= MV) (± standard deviation = SD) was 43.80 (± 27.87) processing days, whereby the variation was wide as indicated by the SD. Table 3 shows the participation rate and the number of data analyses per case after sorting according to the criteria, indicating that the most data of differentiation were excluded from case 3, followed by case 5. There was no case where the dropout rate was remarkably high, so the decrease was consistent.

**Table 3:** Number of participants attending the respective cases during the course and number of evaluated datasets that fulfilled the criteria for analysis. In cases of empty fields, the task was not required in the particular case.

**Case**		**1**	**2**	**3**	**4**	**5**	**6**	**7**	**8**	**9**	**10**
**Number of participations**		247	247	247	247	246	244	242	240	239	237
**Number of datasets in analysis**	Differentiation	207	226	190	218	204	210	226	219	213	223
	Calculation of dWBC		212	228	230	242	237	235	237	236	233
	Calculation of EI		235	238	244		239	240	239	238	234

#### 3.1.1. Cell differentiation

In total, the students viewed and morphologically assigned 1033 cell images each. Considering the results of the success rates of the students, the results were in the upper, i.e., successful range in all cases. Figure 3 shows the success rates of the cases. In total, the minimum was 0.39 (case 3) and maximum 1.00 (all cases except case 3 and case 5), i.e. all cells correctly identified. With a few individual exceptions, the pass mark was achieved throughout. The greatest dispersion of results was observed in MV (± SD) in case 3 with 0.855 (± 0.078) and case 5 with 0.873 (± 0.056), while the students achieved the best differentiation results in case 2 with 0.991 (± 0.009). In case 8, a slight decrease in the results with 0.953 (± 0.036) can be observed, while the students achieved the second best result in case 10 with 0.984 (± 0.018).

**Figure 4:**
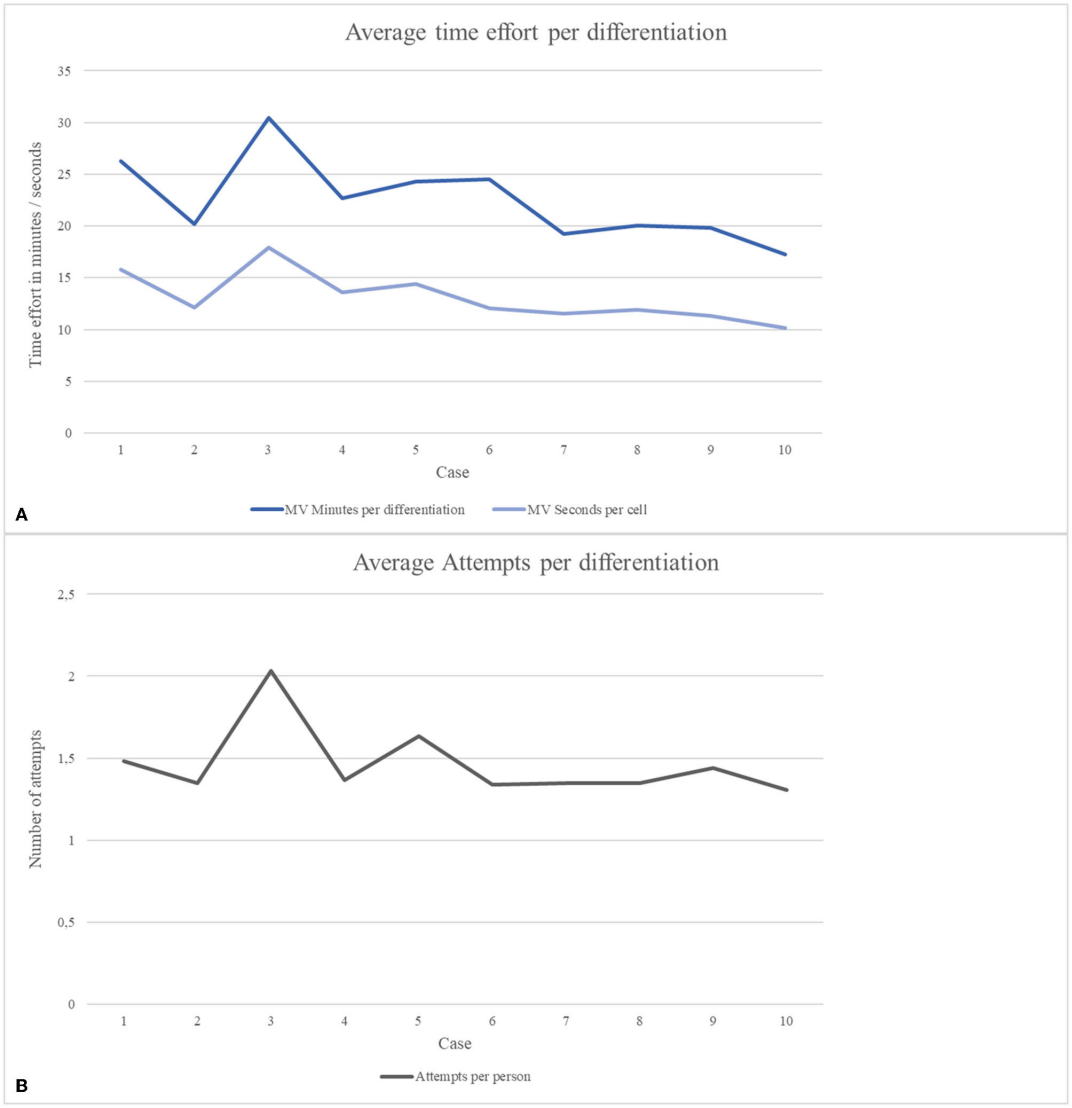
Average results of processing time afforded for differentiation tasks and the total number of started attempts in each case. **(A)** Development of average processing time in minutes per differentiation and converted to seconds spent on a single cell. **(B)** Number of started attempts per differentiation of each case.

On average, a time period of 22.48 min was needed per differentiation. Students improved from an initial 26.27 min for differentiation of 100 cells in case 1 to a total of 17.27 min in case 10. Figure 4 shows the development of the minutes spent per case and the seconds spent in relation to the individual cells per case. It can be seen that the development is not linear, but that a conspicuously higher amount of time was spent in case 3 (30.41 min/17.89 s per cell) and case 5 (24.29 min/14.43 s per cell). The total number of attempts were counted per student and case. Also, MVs were determined (Figure 4). While the students spent an average of 1.48 attempts on differentiation in case 1, in case 10, the average was 1.30 attempts. There was no remarkable improvement during the MC, but Figure 4 shows that in case 3 and case 5, with 2.03 and 1.63 attempts, respectively, more attempts were made on average than in the rest of the MC.

#### 3.1.2. Calculation of dWBC

The mean success rate when calculating the dWBCs achieved by the participants resulted in 0.893 (± 0.198), with a minimum of 0.789 (± 0.271) in case 3 and a maximum of 0.930 (± 0.146) in case 10. As a calculation task consisted of six single tasks in case 2 and eight single tasks in all other cases, the processing time was examined as the time spent on a single calculation task with an average result of 26.62 (± 25.08) s. The mean changed from 52.70 (± 43.9) s in case 2 to 20.03 (± 17.29) s in case 10. On average, the students spent 1.38 (± 0.58) attempts on a dWBC calculation. In case 2, the average was 1.6 (± 0.66) attempts; in case 10 it was 1.36 (± 0.56) attempts.

#### 3.1.3. Calculation of EI

The mean success rate resulted in an MV (± SD) of 0.78 (± 0.30), with the lowest mean rate of 0.562 (± 0.322) in case 2 and a maximum of 0.891 (± 0.891) in case 9. Three single tasks were required here for all calculations, which is why the minutes per task can be given as processing time. The mean calculation time improved from 4.55 (± 3.63) min in case 2 to 1.52 (± 1.26) s in case 10. On average, the students spent 1.5 (± 0.6) attempts on a calculation. In case 2, the average was 1.87 (± 0.59) attempts. In case 10, it was 1.31 (± 0.55) attempts.

### 3.2. Feedback results

Of a total of 247 students who participated in the MC, 192 responded in the voluntary feedback at the end of the course (response rate 78%). With 48 and 33 responses for both open-text questions, the participation was lower than in the 12 rating questions.

#### 3.2.1. Rating scale

**Figure 5:**
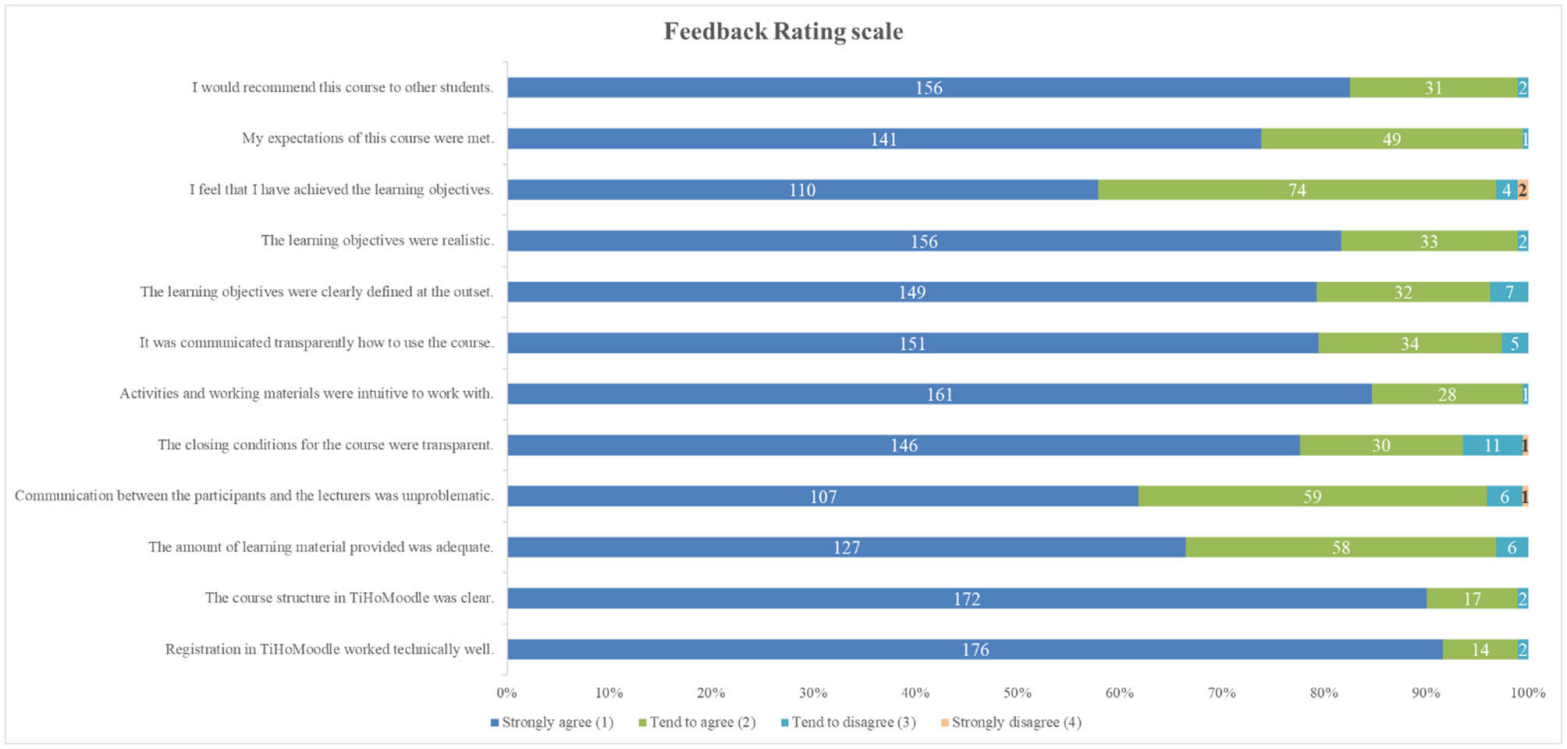
Results of feedback ratings in absolute numbers. The statements rated by the participants are listed. Blue and green bars illustrate numbers of rating “1” or “2”, representative of positive rating of the statements, while turquoise and orange bars illustrate numbers of rating “3” or “4”, representative of negative ratings of statements (see key to symbols).

Since the rating questions were not fully completed by all 192 participants (see Table 4), 188.67 ± 4.87 responses per question were documented. Table 4 shows the individual statements rated by the students including the participation, while Figure 5 illustrates the results of all rating questions in particular. Ratings ranged from score 1 = “strongly agree” to score 4 = “strongly disagree”. It is noticeable that all rating questions were predominantly rated positively, i.e., with score 1 or 2. Overall, the ratings ranged from 1.09 ± 0.33 to 1.46 ± 0.60. Of the total 2,264 awarded scores, score 4 (“strongly disagree”) was awarded four (= 0.18%) times and score 3 (“tend to disagree”) 49 (= 2.16%) times in total. The first two technical questions received the best ratings with mean scores of 1.09 ± 0.33 (“The registration in TiHo Moodle worked properly from a technical point of view”) and 1.11 ± 0.35 (“The course structure in TiHo Moodle was clear”). In terms of questions dealing with the general conditions set by the lecturers, the questions “The activities and work materials were intuitive to use” and “The learning objectives were realistic” were rated particularly well with 1.16 ± 0.38 and 1.19 ± 0.42, respectively. Of the last questions including the personal ratings, “I would recommend this course to other students” achieved the best result with 1.19 ± 0.42, which is of particular interest in our case. The questions with the lowest scoring results were “The communication between the participants and the lecturers was unproblematic” with 1.43 ± 0.59 and “I feel that I have achieved the learning objectives” with 1.46 ± 0.6, which were still rated positively.

**Table 4:** Twelve statements to be rated forming the first part of the feedback module.

**Categories of statement**	**Statements asking for feedback**	**Number of responses**	**Mean rating score**
Technical aspects	The registration in TiHoMoodle worked properly from a technical point of view.	192	1.09
	The course structure in TiHoMoodle was clear.	191	1.11
Teaching quality	The amount of learning material provided (scripts and videos) was adequate.	191	1.39
	The communication between the participants and the lecturers was unproblematic.	173	1.43
	The closing conditions for the course were transparent.	188	1.29
	The activities and working materials were intuitive to work with.	190	1.16
	It was communicated transparently how to use the course.	190	1.23
	The learning objectives were clearly defined at the outset.	188	1.24
	The learning objectives were realistic.	191	1.19
Personal perception	I feel that I have achieved the learning objectives.	190	1.46
	My expectations of this course were met.	191	1.27
	I would recommend this course to other students.	189	1.19

Not all statements achieved the same number of responses.

The assigned score is the average of each statement, as “Strongly agree” = 1, “Tend to agree” = 2, “Tend to disagree” = 3, and “Strongly disagree” = 4.

#### 3.2.2. Open-text answers

The question asking for suggestions for improvement was answered by 48 students (19.43% of all course participants). The wish for further explanations on the assessment of erythrocyte morphology in the course itself was expressed particularly frequently (23 responses = 47.9% of the suggestions). Eleven responses (= 22.9%) stated that the level of difficulty was high. Eight responses (= 16.7%) criticized the technical presentation of the various contents by mentioning many clicks and scrolls. The suggestion to provide more general information about the course schedule and how the course content is divided between the Moodle course and online lectures was mentioned five times (= 10.4%). Three respondents (= 6.25%) wanted the content to be expanded to include more cell variability and more comments on differentiation. The wish for reference values for the evaluation of red blood cell morphologies was also mentioned three times (= 6.25%). The remaining individual annotations included the correction of content mistakes, more variety in cells, and a greater offer of attendance dates.

The final free-text question, “further comments”, allowed students to comment on the Moodle course in any way and was responded to by 33 students (13.36% of all course participants). Responses were generally positive, with 29 (87.9%) of the comments containing overall positive statements about the Moodle course itself. Fourteen of these explicitly referred to perceived learning success (42.4% of the responses). In the category of personal perceptions, eight statements referred to “fun” and another eight found the content of the course “interesting” (24.24% each). Seven responses complimented the structure of the course. Points of criticism in this context were the general course information (two comments) and the wish for an optimized course adaptation (three comments) as well as further explanations (two comments).

## 4. Discussion

### 4.1. Course concept

The main purpose of the study was to introduce the MC as one possible method of teaching hematological content in a digitalized form by presenting the changes the students showed in the processing results in addition to the student's perception of this kind of replacement of microscopy exercises. Overall, we found a high acceptance for the digitized format combined with online lectures and the highly reduced attendance hours working on microscopes of the CPC. This was reflected in the high attendance rate, since it was a voluntary supplementary offer for students who participated in all the remaining attendance hours of the CPC. The dropout rate during the course was generally low, at 4% in total. Of the students, 93 (37.65% of all starters) repeated the contents of the MC by reworking on some of the cases after completing the final case. Since the course was conducted shortly before the final semester exams, we assume that the content could also have been repeated for practicing purposes. The use of virtual patients via Moodle provided the opportunity to familiarize the participants with the routine of differentiating cells according to morphological criteria. In this way, the practical relevance of the content by means of clinical examples can be illustrated and the content taught is more interesting for the students, as shown through the results of their feedback. TiHoMoodle as the chosen platform itself overall proved to be beneficial for this course when structuring the clinical cases in a clear arrangement. This was also confirmed by feedback comments of participants. The compilation of individual modules was a way to provide information step by step in different cases. Features of digitized teaching formats that positively influence learning success are also described in the literature. In 2012, Kotzer et al. described this approach as promoting motivation through explorative and independent learning of the course contents (28). The study refers to Brandl's description of Moodle being grounded in a socio-constructivist theory of learning (29) and describes an increase in motivation due to self-determined action based on more personal responsibility, using learning methods such as modules on platforms like Moodle. The evolution toward digitized teaching formats in veterinary medicine has generally been investigated in a review of digital teaching formats using mini-meta-analysis. In 2022, Muca et al. found a wide variability of different tools for handling digital teaching formats due to contact restrictions caused by the COVID-19 pandemic. They particularly highlighted the increasing use of portable electronic devices for the learning process (30). We also consider it advantageous to let the students work according to their own schedule, as the strongly varying number of processing days shows that the students followed very different time management strategies when processing the cases. Hildebrandt et al. described the lack of flexibility in traditional teaching formats as a fundamental problem according to survey results on students in veterinary medicine in Germany (31). In our course, the processing of the cases was sometimes completed in one day (the minimum being 0.3 days) or, at the other extreme, extended to almost the entire activated time period of up to 100 processing days. It also differed widely whether several cases were processed simultaneously or the students worked on only one case, which underlines the benefits of allowing self-regulated time management. Nevertheless, it should be considered that a more precise timing of the cases could have led to better thematic synchronization with the lecture content, since the MC only deepened hematological content of the first course hours and the remaining CPC and lecture hours already thematized other topics of veterinary clinical pathology, e.g., clotting factors or clinical chemistry. Also, individual students proposed optional time limits in the suggestions for improvement in the feedback. However, we believe that the greater flexibility will benefit students more.

Literature on the design of clinical courses as in this case could not be studied in depth before creating the MC, as the outbreak of the coronavirus pandemic could not be predicted. Most of the information and experiences available about digital teaching of hematological contents relate to the field of human medicine. In the context of distance learning, Brueggeman et al. described the use of virtual microscopy to introduce morphological aspects of hematological cells to students in 2012 (32). In that study, they scanned microscope slides under oil immersion to create images, which was later easily managed by the users similar to viewing them through a microscope with different magnifications. This group was compared to students using a traditional microscopy, while the group using a virtual microscopy performed better in subsequent related academic tasks. In this study, working with a microscope was probably better digitally simulated compared to our approach, as virtual microscopy is already used in the fields of pathology, histology, parasitology, and cytology at the TiHo and other institutions for medical education (33). But in contrast to our course, in the study of Brueggeman et al. the students were not offered any face-to-face teaching in which manual microscopy was still learned separately under supervision. Experiences of another specific tool to teach medical knowledge using clinical cases were published in a study by Kraemer et al. in 2005 (34). The training system “3dweb.train” with web functionality was used specifically for teaching hematology and is similar to our approach of creating clinical cases with images of associated blood or bone marrow smears. According to the authors of the mentioned study, the teaching format also received a good response from the participating students. In retrospect, our choice for the Moodle system was obvious, as the students had already worked with Moodle in other subjects and the learning platform had already been integrated in their everyday study routine at our university. Realistic differentiation of peripheral blood smears was meant to be achieved by displaying single-cell images, as blood smears are manually differentiated using similar principles of single-cell recognition. The differentiation scheme that the students manually filled out during this process is also used during our clinic routine. Additionally, comments of students in the feedback module frequently confirmed that the blood smear differentiation felt realistic. One point of criticism regarding the technical implementation, however, was the course being structured in a way that each cell was displayed individually and new pages had to be opened via a “next” button for each of the next cells. With at least 100 images per cell differentiation, this generated many extra clicks, which caused unnecessary obstacles depending on the individual terminal device being used.

### 4.2. Results of the casework

In the former CLC, it was difficult to assess how the hematological content was received by individual students, while the MC allowed an assessment of changes in the results of differentiation. A review of internet-based learning in health professions including 206 articles by Cook et al. in 2009 points out a highly positive impact of knowledge and theoretical knowledge on practical skills (35). In our opinion, this is also reflected in the present approach, since high scores in the tasks were achieved by the students and the overall development of their performance showed a slight upward trend as the MC progressed. Since the methods for calculating the dWBCs and the erythrocyte-indices were identical throughout the cases and only the numbers were exchanged, the development of the student's results in these tasks was given less consideration overall in this study in view of the rapidly increased good results. In light of the feedback, it is worth mentioning that wrong results were transmitted due to decimal places, but not based on incorrect calculations. Also, the results of the processing time considering the calculation tasks can be disregarded, since it was not possible to investigate whether the time recorded by the program only reflected the time needed to enter the calculated results. Hence, the results of the leukocyte differentiations were the primary topic of interest.

The difficulty of the cases in general does not show a linear increase. This became visible by the different observations in cases 3 and 5, where lower success results, higher dropout rates, more time required, and more attempts occurred on average (Figures 3, 4, Table 3). Table 1 shows that with metamyelocytes, myelocytes, and myeloblasts, cells were presented, which are rarely found in peripheral blood smears making the cases more difficult. In our opinion, these two cases can be compared with each other, as similar cell morphologies were treated and an average improvement in all mentioned study objectives was observed from case 3 to case 5. Therefore, we also consider it appropriate not to look at the success rates between the individual cases of the course in a linear form, but to recognize a general change in performance from case 1 to 10. In this process, rates of correct cell differentiation increased slightly. The parallely decreasing processing time (minutes per case and seconds per cell, Figure 4), as the case number increased could provide an association with an increase in competence as these corresponding observations, underlined by statements in the feedback, could suggest an increase in knowledge, self-confidence, and routine. It should be noted that increasing practice in the use of the technical tools may also have had an impact on the improvement of results and time. Reasons for only little change in the results could be the changing animal species and thus the different cell morphologies in the cases, next to changing difficulties of the cases themselves. Regarding the results, it can be noted that the students were able to achieve high results right from the start (Figure 3), which makes it difficult to observe a development as a sign of improvement. We attribute this to the experience gained from the first attendance hour of the CPC, combined with suitable introduction and the range of teaching materials for further reading. With a few exceptions, the pass mark of 80% was consistently achieved and all students received the credits. It can be observed that sometimes a differentiation task was not completed to a minimum of 50% and therefore was not included in the assessment. This occurred 33 times in total, with 12 rejected data sets in case 3 and six rejected data sets in case 5. We may speculate that the students noticed several mistakes through the revision function in the course of an attempt and preferred to restart rather than finish. If the attempt was stopped after 50% of the cells had been processed, we evaluated this as an active decision to submit this attempt and to accept a worse result. These attempts were included in the evaluation, since this scenario occurred rarely. The number of attempts raised in case 3 and 5, but only changed little considered from case 1 to case 10. We relate this to student's improvements of already good attempts once again. Also, it should be noted that no difference was made during analysis as to whether the individual attempts served for a student's own learning purposes or were later repeated as an exercise for the exams at the end of the winter semester. We can only speculate about student intentions.

The way in which a new page had to be opened for each cell during differentiating, which caused many clicks, was one of the most frequently mentioned points of criticism in the feedback in this context. For the re-upload of the subsequent academic year 2022/2023, the display was therefore adjusted in such a way that all cells were displayed on one page below each other. Displaying the correct answer for each cell allowed students to directly monitor the development of their competency themselves. We may assume that microscopy is now simulated more realistically, as the use of a microscope also offers the possibility of reviewing a cell. No other course display settings were changed for the new semester.

The overall results were also slightly skewed, as there were only a few mistakes in the course settings when determining the cells and setting the correct answers. For example, in case 3, one cell had an incorrect answer choice marked as correct, while another cell had two answer options marked as correct instead of one. Since these individual cells barely influence the overall result, we decided to include these individual cells in the evaluation as described. In addition to the selection of mostly characteristic cells, a few cells whose assignment is not entirely clear were also uploaded. Especially in the case of cells representing different developmental stages, such as metamyelocytes, band neutrophils, and segmented neutrophils, there are transitional stages that sometimes cannot be clearly assigned to one or the other cell type. In reality, cells in peripheral blood smears cannot always be clearly assigned or are identified differently by different examiners, where this effect is even more prominent.

When considering changes in student performance over time as measured by time spent on cells and test scores, it is important to keep in mind that the routine gained on technical tools by working on multiple cases at one time can also have a positive impact on scores and processing time compared to working on cases one after another with time gaps. Also, evaluating the processing time, it must be taken into account that the authors did not have any information about the technical devices used by the participants. For example, the use of a permanently installed computer with a computer mouse compared to the use of a smartphone can have a significant influence on working quality of the tasks. The participant's respective internet connection must also have had an impact on the processing quality. The same is valid for the results achieved in the tasks. Thus, the use of a smaller screen could have caused more incorrect answers due to poorer overview. Regarding the development of the results during the course, it should be noted that the students were confronted with different mammal species and thus had to assess cells with new morphological characteristics in each case. Despite increasing routine, this led to new difficulties that certainly affected the results. In addition, this study lacks a concrete reference point (e.g., in the form of an exam) at which students' performance in the MC can be directly compared to that in the traditional course format.

## 5. Conclusion

Overall, the results of the study support the interpretation that the MC had a positive impact on the learning success of the students while learning blood cell morphologies. Combined with the findings from the literature mentioned on the development of digital teaching, this highlights the growing importance of incorporating those digital teaching formats in which participants can work more independently, flexibly, and not depending on location. In general, the combination of face-to-face sessions for learning laboratory methods with digital self-study can be seen as very beneficial. In this way, as in our case, practicing laboratory procedures could still be supervised by experienced laboratory staff. At the same time, students have the flexibility to learn cell morphologies using illustrative clinical cases and practice cell differentiation in routine analyses at their own pace with immediate feedback available if the cell was recognized correctly.

## 6. Outlook

A recent survey among veterinarians and students in Germany showed that clinical pathology is rated as being of high relevance for the future practice of the profession (36). In view of the increasing role of digitalization in medical education due to technical developments and a growing affinity of new generations for multimedia devices and innovations, the transfer of digital formats to hematological training programs in veterinary medicine offers great potential. In addition, the effort required for microscopic photography of blood smears is greatly simplified by newer technologies such as the digitization of entire sets by scanning and automatically assigning the individual cell morphologies per image recognition. In this way, databases of blood smears from a wide variety of animal species could be created, archived, and continuously expanded to be applied in teaching. In future similar studies, it would be beneficial to identify participants' choice of technical device to examine completion time and understanding of course structure more reliable. The high grade of acceptance of the MC and the positive feedback led to the same course being offered to subsequent students in 2022/2023 with some improvements but using the same course concept. Which difficulties arise from the individual cell morphologies, also in relation to the different animal species, will be the subject of another study.

## Author's note

Parts of this study have already been presented at a conference and are therefore available as conference abstract: 31st Annual Meeting of the Section Internal Medicine and Clinical Laboratory Diagnostics (InnLab), DVG (Deutsche Veterinärmedizinische Gesellschaft e.V.), 02-03-2023, Göttingen, Germany.

## Data availability statement

The raw data supporting the conclusions of this article will be made available by the authors, without undue reservation.

## Ethics statement

Ethical approval was not required for the studies involving humans because as the participants agreed that their data could be processed and the analysis of it could be carried out, and their personal identity was protected by anonymization, it was not necessary to call in an Ethics Committee. The data protection declaration had previously been checked by the university's legal protection advisor. The studies were conducted in accordance with the local legislation and institutional requirements. The participants provided their written informed consent to participate in this study.

## Author contributions

HM analyzed the data and wrote the manuscript. MW planned and supervised the study and created the Moodle-course. MW and MG laid internist basics for the Moodle course. MG as head of the laboratory, provided the materials to work with. ES, JZ, and HM analyzed and categorized the feedback data. FK helped with analyzation and interpretation of statistics. All authors contributed to manuscript revision, read, and approved the submitted version.
